# Microfungi in Drinking Water: The Role of the Frog *Litoria caerulea*

**DOI:** 10.3390/ijerph7083225

**Published:** 2010-08-19

**Authors:** Noel B. Sammon, Keith M. Harrower, Larelle D. Fabbro, Rob H. Reed

**Affiliations:** 1 Centre for Plant and Water Science, CQUniversity Australia, Bruce Highway, Rockhampton, Queensland, Australia 4702; E-Mail: r.reed@cqu.edu.au (R.H.R.); 2 Centre for Environmental Management, CQUniversity Australia, Bruce Highway, Rockhampton, Queensland, Australia 4702; E-Mail: l.fabbro@cqu.edu.au (L.D.F.)

**Keywords:** filamentous fungi, yeasts/yeast-like, Australia

## Abstract

Microfungi were recovered from all parts of a municipal water distribution system in sub-tropical Australia even though virtually no colony-forming units were recovered from the treated water as it left the treatment plant. A study was then undertaken to determine the potential sources of the microfungal population in the distribution system. Observation of frogs (*Litoria caerulea*) using the internal infrastructure of a reservoir as diurnal sleeping places, together with observation of visible microfungal growth on their faecal pellets, led to an investigation of the possible involvement of this animal. Old faecal pellets were collected and sporulating fungal colonies growing on their surfaces were identified. Fresh faecal pellets were collected and analysed for microfungal content, and skin swabs were analysed for yeasts. It was found that the faeces and skin of *L. caerulea* carried large numbers of yeasts as well as spores of various filamentous fungal genera. While there are many possible sources of microfungal contamination of municipal drinking water supplies, this study has revealed that the Australian green tree frog *L. caerulea* is one of the important sources of filamentous microfungi and yeasts in water storage reservoirs in sub-tropical Australia where the animal is endemic.

## 1. Introduction

An extensive identification and enumeration study of microfungi in a treated municipal water supply system in sub-tropical Australia over an 18 month period showed that microfungi are present throughout the system [1]. Analysis of the treated water leaving the single water treatment plant indicated that the treatment processes are very effective in removing or inactivating microfungal propagules since only occasional microfungal colony-forming units (CFU) were recovered from samples taken at the treatment plant outlet. However, microfungal CFU were routinely recovered from treated water storage reservoirs and distribution mains throughout the distribution system.

**Figure 1.**
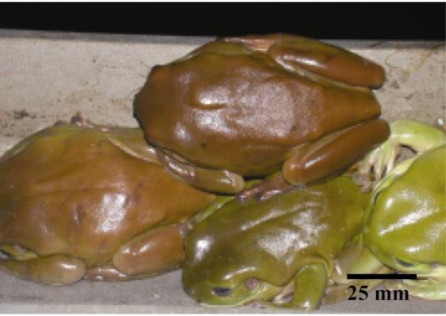
Group of *Litorea caerulea* specimens asleep on a side rail of an internal stairway in a reservoir.

This finding led to a further investigation to attempt to identify the source(s) of such post-treatment microfungal population. During the collection of monthly water samples from one of the roofed storage reservoirs, designated R3, over an 18 month period it was noticed that numbers of the large Australian green tree frog, *Litoria caerulea*, were always resident within the reservoir, occupying the internal infrastructure of stairway, supporting columns and roofing beams as diurnal sleeping places. They particularly favoured the sloping C-section side rails of the stairway (Figure 1).

Frog excrement was also evident on these structures but it was clear that most faecal pellets are deposited directly into the water or are dislodged into the water by movement of the animals. During the study period a number of unexplained cases of low *Escherichia coli* counts were reported by a number of water authorities throughout Queensland. The regular observation of *L. caerulea* within water storage reservoir R3 in the water supply system under study led to the hypothesis that the source of those *E. coli* counts was the excrement of *L. caerulea.* Subsequent investigation demonstrated that the frog *L. caerulea* carries very large *E. coli* loads [2]. The water authority concerned has since taken steps to exclude *L. caerulea* from the reservoirs in its water distribution system.

Concurrently, it was noticed that old faecal pellets of *L. caerulea* which remained on the internal structures of reservoir R3 were showing evidence of extensive microfungal growth on their surfaces. This observation suggested three possibilities; (i) that frog excrement contained viable microfungal propagules which germinated post-voidance, (ii) that frog excrement acted as a suitable substrate for the germination and growth of airborne spores which landed on it, or (iii) both. This led to an investigation which aimed to determine whether or not the Australian green tree frog *L. caerulea* may be one of the sources of the microfungal propagules recovered from the mains and reservoir water during the identification and enumeration study mentioned earlier.

## 2. Materials and Methods

Samples were collected from the interior stairway and accessible structures of a roofed water service reservoir (R3) in a city in Central Queensland, Australia.

### 2.1. The Reservoir

Reservoir R3, which was the site for this study, is eminently suitable for that purpose. Its walls and floor are cast concrete and it is roofed with galvanised sheeting on galvanised beams supported by concrete columns. It has a maximum capacity of *ca*. 10.2 megalitres and is filled solely by an underground trunk main fed directly from another reservoir, designated R2, located *ca*. 2 km away. Reservoir R2 is automatically re-chlorinated to *ca*. 0.60 ppm. During the 18 month period over which the identification and enumeration study was conducted the average recovery of yeasts from reservoir R2 was <1 cell per litre and filamentous microfungi was only 3 cells per litre. Consequently the most likely explanation for the existence of the microfungal population in reservoir R3 was through introduction of propagules at the reservoir itself. Reservoir R3 was not re-chlorinated at the time of this study, and free chlorine readings during the identification and enumeration study period averaged less than 0.10 ppm.

### 2.2. Surface Growth of Microfungi

Twenty aged faecal pellets of the Australian green tree frog *L. caerulea* were collected from the internal stairway and accessible infrastructure of reservoir R3. These were placed in individual sterile 30 mL polycarbonate screw-top tubes (Sarstedt) which were capped immediately after each sample was collected. At the laboratory, the surfaces of the pellets were examined for the presence of microfungal colonies under a Wild M3Z stereomicroscope. Fragments of sporulating colonies were then picked with a dissecting needle from the surfaces of five randomly selected pellets. These fragments were placed in drops of fuchsin acid/lactic acid stain (0.1 g acid fuchsin in 100 mL lactic acid) on glass microscope slides and examined under a Leica DMLB light microscope. Sporulating microfungal colonies were identified by their reproductive structures and spores to genus level by reference to Carmichael *et al*. [3], Ellis [4,5], Gilman [6], and Kendrick and Carmichael [7].

### 2.3. Microfungal Analysis of Frog Excrement

Ten freshly deposited *L. caerulea* faecal pellets were collected for further microfungal analysis. Each pellet was weighed then carefully broken apart and a needle-point sample was taken from the interior of the pellet, placed in a drop of acid fuchsin/lactic acid stain on a glass microscope slide, and examined under a Leica DMLB light microscope for visual evidence of the presence of microfungal spores. Each pellet was then placed in a sterile 30 mL polycarbonate screw-top tube (Sarstedt), 20 mL of sterile reverse osmosis water (SROW) was added, and a faecal suspension was produced by mixing the contents thoroughly for 1 minute on a CSV90 Auto vortex mixer at maximum setting No. 8. Each suspension was filtered through sterile muslin and the filtrate was brought up to 100 mL with SROW. Streptomycin was added to the filtrate at the rate of 100 mg L^−1^ to inhibit bacteria, and decimal dilution series to 10^−3^ were prepared from the filtrate using SROW. An aliquot of 100 μL of each dilution was spread onto malt extract agar amended with chloramphenicol (MEAC) (malt extract 10 g, glucose 10 g, bacteriological agar 10 g, peptone 0.5 g, reverse osmosis water 500 mL, chloramphenicol 50 mg) in standard plastic Petri dishes. The culture plates were surface-dried in a biological safety cabinet for 1 h immediately before use. The spread plates were incubated at 25 °C in the dark. Yeast and filamentous fungal colonies were counted over 7 days and the estimated number of CFU g^−1^ of frog excrement was calculated. Filamentous colonies were identified to genus level and the estimated percentage of each genus recovered was calculated.

This procedure was repeated on another five fresh faecal pellets of *L. caerulea* collected in September/October 2009 after several months of extremely dry, low-humidity weather. Ten yeast colonies, which appeared to be different to each other in terms of gross colony morphologies and which were represented in all of the samples, were picked from the cultures and sub-cultured. These were sent to Sullivan Nicolaides Pathology Laboratories in Rockhampton, Queensland for identification.

### 2.4. Skin Swabs—Yeast

An area of 9 cm^2^ of the dorsal skin of each of 10 sleeping adult specimens of *L. caerulea* (CQUniversity Animal Ethics permit No. A10/04-258) was gently swabbed to determine if yeasts were endemic on the skin of these animals. A sterile cotton bud, with long wooden handle, dipped in SROW was used for this purpose. The cotton bud was then placed in a sterile 30 mL polycarbonate screw-top tube (Sarstedt) containing 10 mL of SROW, the wooden handle of the cotton bud was broken off, and the tube was capped. Sterile rubber gloves were worn to prevent accidental cross-contamination between operator and frog. The tubes were transported back to the laboratory in pre-cooled containers and were processed immediately on arrival. Each tube was vortexed on a CSV90 Auto vortex mixer at setting 8 for 30 sec. and the swab was discarded after expressing excess solution by pressing it against the side of the tube. A decimal dilution series to 10^−3^ was prepared from the contents of each of the ten tubes using SROW. Spread plates were prepared using MEAC culture plates which had been surface-dried in a biological safety cabinet for 1 hr. immediately before use. An aliquot of 200 μL of each of the dilution series was pipetted onto these plates and spread with sterile plastic spreaders. The culture plates were incubated in the dark at 25 °C and yeast colonies were counted daily for 3 days. Ten yeast colonies, which appeared to be different to each other in terms of gross colony morphologies and which were represented in all of the samples, were picked from the cultures and sub-cultured. These were sent to Sullivan Nicolaides Pathology Laboratories in Rockhampton, Queensland for identification.

### 2.5. Photographs and Photomicrographs

Photographs were taken with a Nikon Coolpix 995 digital camera. Photomicrographs were taken with an Olympus Colorview III digital camera mounted on a Leica DMLB light microscope.

## 3. Results

### 3.1. Surface Fungal Growth

**Figure 2.**
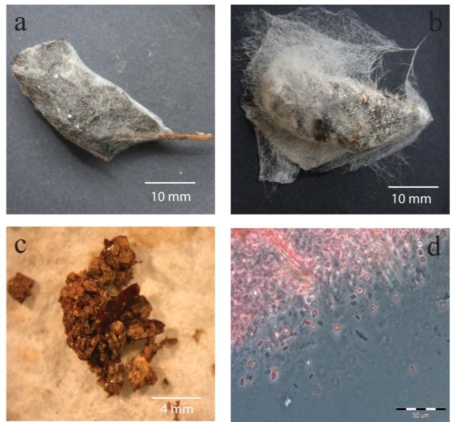
**(a,b)** Two aged faecal pellets of *Litoria caerulea* covered with extensive growth of sporulating microfungi. **(c)** Soil particles from a faecal pellet of *Litoria caerulea*. **(d)** Microfungal spores from a needle-point fragment of the interior of a fresh faecal pellet of *Litoria caerulea* showing visible microfungal spores.

All 20 aged faecal pellets collected and examined for surface fungal growth supported extensive, visible, sporulating microfungal colonies (Figure 2a,b). The genera identified on the five pellets selected at random for colony characterisation included *Aspergillus* (three species), *Penicillium* (two species), *Absidia, Memnoniella, Stilbella, Fusarium, Acremonium, Syncephalastrum* and *Geotrichum*.

### 3.2. Microfungi Recovered from Excrement

Most of the faecal pellets from which needle-point samples were taken and examined microscopically in May 2009 contained visible microfungal spores (Figure 2d). Many of the faecal pellets contained visible grass fragments and soil particles (Figure 2a,c).

The estimated CFU per gram of excrement and characterisation of filamentous microfungi and yeasts, based on the colony recoveries from the decimal dilution series made from the 10 pellets collected in May 2009 and the five collected in September, 2009 are in Table 1.

### 3.3. Yeast Identification—Excrement

The 10 yeast isolates sub-cultured from colonies recovered from the five frog pellets collected in Sept/Oct 2009 were identified as 1 *Rhodotorula* sp., 6 *Trichosporon* spp., 1 *Candida* sp., 1 *Candida famata*, and 1 *Candida glabrata*.

### 3.4. Yeasts Recovered from Skin Swabs

The skin swabs from all 10 specimens of *L. caerulea* tested positive for yeasts. The estimated CFU per cm^2^ of dorsal skin, based on the CFU recovered from the decimal dilution series made from the 10 swab samples collected in May 2010 are shown in Table 2. The 10 yeast isolates sub-cultured from colonies recovered from the skin swabs were identified as 3 *Trichosporon asahii*, 3 *Trichosporon* spp., 1 *Rhodotorula* sp., 1 *Aureobasidium pullulans*, 1 *Candida* sp. and 1 unknown species.

It was notable that the yeast populations recovered from several of the frogs showed a dominance of one species and the dominant species varied from frog to frog.

## 4. Discussion

The large green tree frog *L. caerulea* is endemic across the northern half of Australia and parts of Papua New Guinea [8,9]. In its natural habitat this nocturnal, arboreal species hides during the day in hollow tree limbs, in dense foliage, and in similar protected places. It also has an affinity for human dwellings and other man-made structures including roofed water storage reservoirs as daytime hiding places, and seems to prefer these to its natural habitat. These structures provide a dark, warm, humid atmosphere, close proximity to water, and protection from predators, conditions which are ideal for this animal.

On every occasion that reservoir R3 was visited for water sampling purposes, mature specimens of *L. caerulea* were always found sleeping on the internal stairway, adjacent roof superstructure, and supporting columns within the reservoir. The suction pads on the toes of these animals enable them to easily climb vertical surfaces. At night this ambush predator comes to ground to prey on coleopterans and other insects as well as small vertebrates. The prey is engulfed, secured with its sticky tongue and vomerine teeth, and swallowed whole. As a consequence of this ground-feeding method the frog frequently ingests dried grass fragments and soil particles along with its prey, and these were commonly observed in the faecal pellets (Figure 2 a,c). These materials are natural substrates for many of the saprobic microfungi which were recovered from frog faecal pellets and also from water samples taken from the water body in the reservoirs [1]. Consequently it would be expected that such debris would introduce microfungal spores into the frog’s digestive system. Furthermore, many of the frog’s prey species are known to carry entomopathogenic fungi such as *Paecilomyces* spp. [10] which are also found in soil and on plants. The large number of fungal spores observed in needle-point samples of fresh frog faecal pellets also supports the theory that fungal spores are ingested with prey and extraneous debris, and pass through the animal’s digestive system unharmed.

It is clear that in areas of Australia where *L. caerulea* is endemic, the species is a potential source of the microfungal population of drinking water systems, and this could occur in two ways. Firstly by introduction of fungal spores from outside the reservoir in the frog’s faeces which are, in most cases, voided directly into the water body of the reservoir. The frog’s ground-feeding method ensures ingestion of extraneous material along with spore-laden prey and it is clear from the data that many of those spores survive the frog’s digestive system. This is supported by the variety of filamentous microfungal genera recovered from the frog pellets and the occasional large percentages of individual genera. For example, Table 1 shows that 97.7% of microfungal CFU recovered from frog pellet #2 was *Paecilomyces*, a known entomopathogenic genus. Samples #4 and #11 carried high percentages of *Penicillium* spp. while 45.1% of the filamentous fungi CFU recovered from sample #8 were *Aspergillus* spp. These genera are saprobes of plant debris and are also found in soil. The data in Table 3 also suggests that the frog’s digestive system naturally supports very high yeast populations. With the sole exception of sample #2, high numbers of yeasts were recovered from all of the pellets tested. It is notable that yeast and yeast-like colonies were frequently recovered from water samples collected from reservoir R3 during the separate identification and enumeration investigation [1]. Secondly, when excrement is deposited onto the internal superstructure above the maximum water level it provides a substrate where microfungi grow and sporulate thus adding further to the airborne spore load within the reservoir and subsequently in the water body.

The high prevalence and number of yeasts and yeast-like microfungi recovered from frog excrement and from skin swabs suggests that these microorganisms are resident commensals of the gut and skin of these animals. These high numbers, together with the knowledge that the animal carries high *E. coli* loads, suggests that *L. caerulea* should be excluded from reservoirs wherever possible and that adequate residual free chlorine levels should be maintained at all times. It is noteworthy that during the identification and enumeration study, 18 monthly water samples from reservoir R3, which was not rechlorinated during that study, produced an average of 147 yeast cells per litre of water. The water utility involved in this study has already taken appropriate action, and is constructing a rechlorination facility at reservoir R3 to manage the risk of recontamination.

The yeast genera *Candida*, *Rhodotorula* and *Trichosporon* which were recovered from excrement of *L. caerulea* collected from reservoir R3 contain species which can be opportunistic human pathogens [11]. Three of the ten representative yeast isolates from frog excrement were *Candida* species. It is known that metabolites of *Candida* are the aetiological agents of candidids, an allergic skin reaction [12]. The recovery of *Candida glabrata* is of some concern since it is a major cause of candidiasis in humans. This yeast, like *C. albicans*, is a commensal organism and is also an opportunistic pathogen causing superficial and systemic infections such as endocarditis, cystitis, osteomylitis, vaginitis and fungemia [13]. *C. albicans* was not found in this study but has been recovered from overseas water systems [14]. Thuraisingam and Denning [15] reported increasing frequency of systemic and vaginal infections with *C. glabrata* and stated that such infections are difficult to treat. Vaginitis is caused by both *C. albicans* and *C. glabrata*. However, increased prevalence of *C. glabrata* in diabetic women was reported by Ray *et al*. [16] who isolated *C. glabrata* from 61.3% and *C. albicans* from only 28.8% of 111 consecutive diabetic patients suffering from vulvovaginal candidiasis during their study into the prevalence of *C. glabrata* and its response to certain treatments. According to Gugic *et al*. [17], *C. glabrata* is the second most common species of *Candida* found in hospital patients in the U.S.A.

The other yeasts recovered from frog excrement and skin, *Rhodotorula* spp. and *Trichosporon* spp., are commonly occurring genera and while they have, on rare occasions, been associated with opportunistic human infections their involvement is of little importance [12].

It is not known if infections and allergies caused by *C. glabrata* or other yeasts can be acquired through contact with water containing them, but it is reasonable to expect that this may be so. In recent years it has been increasingly recognised that opportunistic, pathogenic microfungi, particularly filamentous genera such as *Aspergillus*, may be the waterborne aetiological agents of nosocomial mycoses but only a limited number of studies have been undertaken into this potential mode of infection.

## 5. Conclusions

The evidence presented in this study suggests that the Australian green tree frog *L. caerulea*, a diurnal resident of covered water reservoirs, is a source of yeast/yeast-like and filamentous microfungi found in water supply systems in northern Australia.

The excrement of this species introduces fungal spores directly into the water body of water storage reservoirs and, when deposited on internal reservoir architecture, it also acts as a substrate on which microfungi grow and sporulate thus increasing the spore load within the reservoir air and consequently in the water body. The results presented here suggest that the frog species *L. caerulea* is an important source of yeasts in water storage reservoirs in areas where the animal is endemic.

While this study was focused on a municipal water supply system where the water is treated and water quality is monitored closely, the findings would also be applicable to household water supply derived from rainwater tanks. In fact, if *L. caerulea* can gain access to domestic rainwater tanks to which exclusion devices are not fitted then a potential health problem could very well exist. Domestic rainwater tanks in Australia are rarely, if ever, chlorinated and the frog to water volume ratio is likely to be high because of the relatively small water-holding capacity of such tanks.

## Figures and Tables

**Table 1.** Composition of microfungal CFU recovered from *Litoria caerulea* excrement.

Sample No.	Total CFU g^−1^ of excretum	*Penicillium*	*Paecilomyces*	*Aspergillus*	*Cladosporium*	Other f/fungi incl. asporous	Yeasts and yeast-like
		
		Percentage of total					
May 2009							
1	15.2 × 10^6^	1.1					98.9
2	4.3 × 10^6^	2.3	97.7				0.0
3	30.6 × 10^6^						100.0
4	9.5 × 10^6^	46.3	9.3			24.0	20.4
5	11.4 × 10^6^	3.9					96.1
6	4.4 × 10^4^	9.9		3.9		5.0	81.2
7	7.8 × 10^6^						100.0
8	1.2 × 10^5^			45.1	0.6	0.5	53.8
9	3.2 × 10^6^	3.2		9.8	2.2		84.8
10	22.1 × 10^6^						100.0
Sept/Oct 2009							
11	6.5 × 10^5^	20.3					79.7
12	4.0 × 10^6^	1.0				3.8	95.2
13	44.0 × 10^6^						100.0
14	1.3 × 10^5^	6.5	3.3	6.6		8.2	75.4
15	4.2 × 10^6^	4.2	4.2				91.6

Average	10.5 × 10^6^	6.6	7.6	4.4	0.2	2.8	78.5

**Table 2.** Yeast CFU recovered from *Litoria caerulea* skin swabs.

Sample No.	1	2	3	4	5	6	7	8	9	10	Average
Yeast CFU per cm^2^ of skin	956	211	617	228	978	133	300	2,167	639	1,333	623
